# Towards a Novel Class of Multitarget-Directed Ligands: Dual P2X7–NMDA Receptor Antagonists

**DOI:** 10.3390/molecules23010230

**Published:** 2018-01-21

**Authors:** Olga Karoutzou, Seung-Hwa Kwak, So-Deok Lee, Daina Martínez-Falguera, Francesc X. Sureda, Santiago Vázquez, Yong-Chul Kim, Marta Barniol-Xicota

**Affiliations:** 1 Laboratori de Química Farmacèutica (Unitat Associada al CSIC), Facultat de Farmàcia i Ciències de l’Alimentació, and Institute of Biomedicine (IBUB), Universitat de Barcelona, Av. Joan XXIII 27–31, E-08028 Barcelona, Spain; olgakarou@gmail.com (O.K.); svazquez@ub.edu (S.V.); 2 School of Life Sciences, Gwangju Institute of Science and Technology (GIST), Cheomdangwagi-ro, Buk-gu, 123, Gwangju 61005, Korea; kwakseunghwa@gmail.com (S.-H.K.); hlwssd@gmail.com (S.-D.L.); 3 Unitat de Farmacologia, Facultat de Medicina i Ciències de la Salut, Universitat Rovira i Virgili, c./St. Llorenç 21, E-43201 Reus, Spain; daina.falguera95@gmail.com (D.M.-F.); francesc.sureda@urv.cat (F.X.S.)

**Keywords:** Alzheimer’s disease, dual target compounds, neuroinflammation, NMDA, P2X7

## Abstract

Multi-target-directed ligands (MTDLs) offer new hope for the treatment of multifactorial complex diseases such as Alzheimer’s Disease (AD). Herein, we present compounds aimed at targeting the NMDA and the P2X7 receptors, which embody a different approach to AD therapy. On one hand, we are seeking to delay neurodegeneration targeting the glutamatergic NMDA receptors; on the other hand, we also aim to reduce neuroinflammation, targeting P2X7 receptors. Although the NMDA receptor is a widely recognized therapeutic target in treating AD, the P2X7 receptor remains largely unexplored for this purpose; therefore, the dual inhibitor presented herein—which is open to further optimization—represents the first member of a new class of MTDLs.

## 1. Introduction

Alzheimer’s disease (AD) is an afflicting neurodegenerative disorder that triggers a progressive loss of neurons and synapses, leading to an irreversible cognitive decline and eventually to death [1,2,3]. The two groups of currently approved anti-AD drugs—cholinesterase inhibitors (donepezil, galantamine, and rivastigmine) [4,5,6] and *N*-methyl-d-aspartate receptor (NMDAR) antagonists (memantine) [7,8]—can, at best, only attenuate AD symptoms for a limited period of time. Therefore, the development of new effective drugs that may halt the neurodegeneration process is urgently needed [9,10,11,12,13].

Although the pathophysiology of AD is still not completely understood, it is widely accepted that the accumulation of extracellular plaques is one of the major hallmarks in AD [14,15,16,17,18,19]. These plaques are mainly composed of beta-amyloid peptide and activated microglia; in the first instance, they produce a neuroprotective inflammatory response to repair damage [20,21].

The ATP-gated P2X7 receptors (P2X7R), widely expressed in the central nervous system (CNS) [22,23,24], have been recently identified as an obligate participant in the activation of microglia [25], where they are especially abundant. Moreover, its physiological activation in other CNS cell lines—as granular neurons—links to calcium-dependent neuroprotective events such as cAMP response element-binding (CREB) activation [26]; this is responsible for protection against glutamate-induced excitotoxicity and Glycogen synthase kinase 3 (GSK3) phosphorylation, which rescues cells from apoptosis [27]. On top of that, P2X7R activation can also trigger the non-amyloidogenic cleavage of the amyloid precursor protein (APP) [28,29]. On the other hand, with higher and chronic levels of ATP exposure—which is characteristic of AD—the P2X7 receptors up-regulate in microglia [30]. This is responsible for catalyzing inflammatory cascades through the release of IL-1β, ATP, and other factors that lead to cell apoptosis. In addition to this, it has been demonstrated that P2X7R also releases glutamate which activates the ionotropic NMDA receptor [31,32,33].

With physiological levels of glutamate, the NMDA receptor is responsible for learning and memory processes [34]; however, the beta amyloid oligomers that are formed in AD reduce glutamate uptake and increase glutamate release, resulting in an elevation of the neurotransmitter levels in the synaptic cleft [35]. This moderate but chronic stimulation of the NMDA receptor provokes excitotoxicity, neuronal loss, and the characteristic decline in memory and cognition observed in AD patients [36]. The clinically approved NMDA receptor antagonist memantine alleviates this process by blocking the NMDAR in a non-competitive manner [37], allowing the correct function of the receptor while preventing excitotoxicity [38]. Although its usefulness has been proved extensively, this drug cannot totally block the neurotoxic cascade of AD.

Taking into account that tackling this complex disease from only one angle seems to be insufficient, in the last few years several groups have started medicinal chemistry programs aimed at designing multi-target-directed ligands (MTDLs) for hitting different biological targets for AD [39,40,41].

Considering the aforementioned role of NMDA and P2X7 receptors in AD, we reasoned that a drug that could target both receptors simultaneously would be highly beneficial as a potential treatment of this disease; it could not only halt its progression but also ameliorate its symptoms. In addition, the similar double-faced neuroprotective–cytotoxic behavior that both receptors display makes them perfect candidates for this purpose. In light of this, the ideal antagonist would be a compound that displayed inhibitory potencies in the low micromolar range, allowing the physiological function of the receptors but, in turn, inhibiting them upon up-regulation or over-activation.

## 2. Results and Discussion

**Figure 1:**
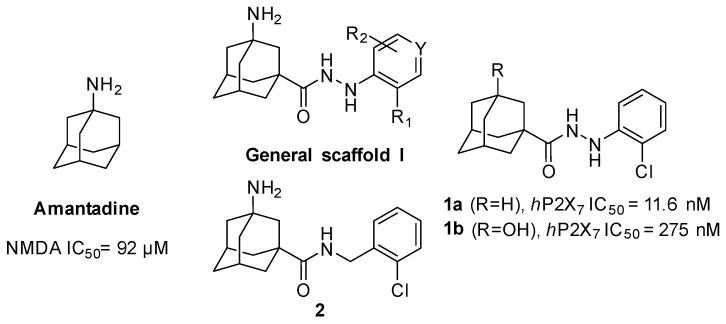
Rational design of the first generation of analogues.

For this, we envisaged the aminoadamantylcarbohydrazides of general structure **I** as putative dual inhibitors (Figure 1). Firstly, **I** features the amantadine scaffold, which is known to be a micromolar NMDAR antagonist (IC_50_ = 92 ± 29 μM) [42]; secondly, the *N’*-arylcarbohydrazide moiety of **1a** is a compound endowed with nanomolar P2X7R antagonist activity (IC_50_ = 11.6 ± 2 nM) [43]. Taking into account the fact that the introduction of a polar alcohol group in **1a** led to a reduction of the antagonist activity of P2X7R (IC_50_ = 275 ± 50 nM for **1b**) [43], we speculated that the compounds of general structure **I** might display low micromolar to high nanomolar P2X7R antagonist activities, thus suiting our aim of designing the first dual NMDAR–P2X7R antagonist with low micromolar activities in both targets.

**Figure 2:**
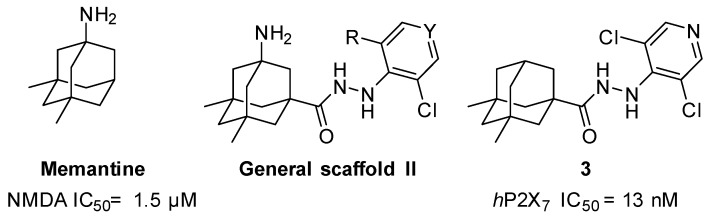
Design of the second generation of antagonists.

In the first instance, seven compounds featuring the general structure **I**, with diverse substitution in the aromatic moiety, were prepared and pharmacologically evaluated as P2X7R and NMDAR antagonists. Compound **2**—which features an amide group—was also prepared and pharmacologically evaluated, with the purpose of assessing the importance of the hydrazide functionality in the linker. Upon testing this batch of derivatives, in order to optimize the activity values obtained (see below), a second generation of dual antagonists was prepared starting from memantine—roughly sixty-fold more potent than amantadine as an NMDAR antagonist (IC_50_ = 1.5 ± 0.1 μM)—and carbohydrazide **3** [44], a known nanomolar P2X7R antagonist (IC_50_ = 13 ± 1 nM) (Figure 2). The aim of moving from general scaffold **I** to **II** was to improve the activity of the compounds as NMDAR antagonists, shown by the previous set of molecules, while preserving the activity against the P2X7 receptor.

**Scheme 1:**
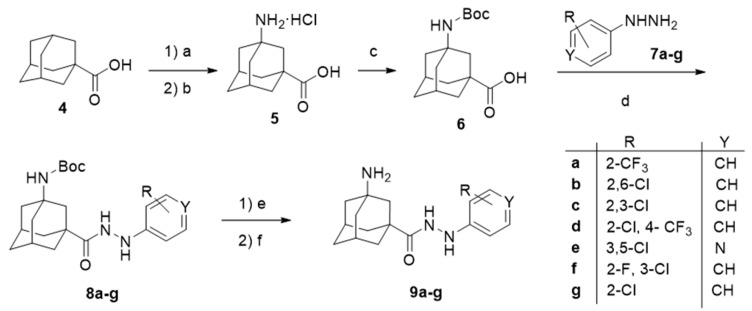
Synthetic route for the first generation of compounds, featuring general scaffold **I**. Reagents and conditions: (a) HNO_3_, H_2_SO_4_, acetonitrile, 0 °C, 88% yield; (b) HCl, H_2_O, reflux, 6 days, 46% yield [45]; (c) (Boc)_2_O, NaOH, THF, 45% yield; (d) **7a–g**, HOBt, EDC, CH_2_Cl_2_; (e) HCl, 1,4-dioxane; (f) NaOH, 14–38% overall yield from **6**.

The synthetic route used to prepare general scaffold **I**, comprehending the derivatives **9a–g** and its analogue **2**, is depicted in Scheme 1. Starting from the commercially available 1-adamantanecarboxylic acid **4**, and following the procedure reported by Schreiner and co-workers, the *N*-Boc protected amino acid **6** was prepared in a good overall yield [45]. In order to explore the structure–activity relationship (SAR) of the aromatic moiety, **6** was coupled, using EDC (1-ethyl-3-(3-dimethylaminopropyl)carbodiimide) and HOBt (1-Hydroxybenzotriazole), with a range of seven aromatic hydrazines, **7a**–**g**, bearing different substituents in the phenyl group. Finally, the desired compounds **9a**–**g** were obtained upon Boc cleavage with hydrochloric acid in 1,4-dioxane. The amide **2**, bearing a modified linker, was accessed following the aforementioned route but reacting **6** with 2-chlorobenzylamine. The pure products **9a–g** and **2**, were fully characterized and pharmacologically evaluated (see below).

**Scheme 2:**
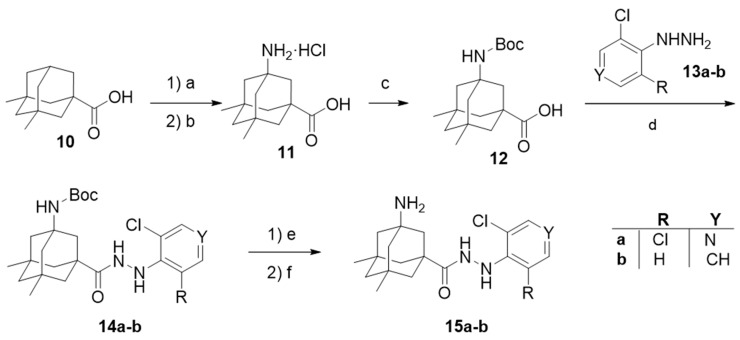
Synthetic route for the second generation of compounds, featuring general scaffold **II**. Reagents and conditions: (a) HNO_3_, fuming H_2_SO_4_, acetonitrile, 0 °C, quantitative yield; (b) HCl, H_2_O, reflux, 6 days, 50% yield [45]; (c) (Boc)_2_O, NaOH, THF, 52% yield; (d) **13a-b**, HOBt, EDC, CH_2_Cl_2_; (e) HCl, 1,4-dioxane; (f) NaOH, 25–28% overall yield from **12**.

Amino acid **11** was prepared using a similar synthetic route, starting from 5,7-dimethyladamantanecarboxylic acid, **10**, but under hardened reaction conditions [45]. After *N*-protection, coupling with 2-chlorophenylhydrazine or 3,5-dichloro-4-hydrazinopyridine, and deprotection, carbohydrazides **15a**–**b** were obtained (Scheme 2).

All new compounds were evaluated for their antagonistic effects on 2’(3’)-*O*-(4-benzoylbenzoyl)-ATP (BzATP)-induced ethidium bromide uptake in human embryonic kidney 293 (HEK 293) cells, stably expressing the human P2X7 receptor, using P2X7 receptor antagonist AZD9056—AstraZeneca’s clinical drug candidate [46]—as a positive control. Inspection of the results shown in Table 1 revealed that, with the sole exception of amide **2**, all the new compounds showed activities in the micromolar range and only the IC_50_ value of **9g** was determined. The comparison of the IC_50_ values of **1a**, **1b**, and **9g** (11.6 nM, 275 nM, and 6.0 μM, respectively) highlights that the introduction of a polar group in the adamantane scaffold is deleterious for the P2X7 antagonist activity and that the presence of the basic amino group is even worse than that of the non-basic alcohol group. However, taking into account that we wanted to design a compound endowed with low micromolar inhibitory potencies against both the P2X7 and NMDA receptors, compound **9g** suited our purpose.

Additionally, to evaluate whether the synthesized compounds were able to antagonize the NMDA receptors, we measured their effects on the increase in intracellular calcium evoked by NMDA (100 μM, in the presence of 10 μM of glycine) on rat-cultured cerebellar granule neurons [47]. All the compounds were only able to inhibit NMDA in the high micromolar range (see Table S1 in the Supplementary Material), indicating that the addition of the phenyl hydrazide moiety is highly detrimental for NMDA activity. The best compound of the series, **9g**, with an IC_50_ = 468 ± 23 μM, was roughly five-fold less potent than amantadine.

Taking into account the fact that the 3,5-dimethyl derivative of amantadine (i.e., memantine) is 60-fold more potent as an NMDA inhibitor than amantadine [48], we envisaged a second series of derivatives featuring the memantine moiety. Hydrazide **3**, featuring two methyl groups in the adamantane scaffold, was reported to be a nanomolar P2X7 inhibitor [44], reinforcing the suitability of this strategy to obtain balanced inhibitory potencies in both receptors. Hence, while the addition of the two methyl groups to the polycyclic scaffold of the adamantane should not be detrimental for P2X7R antagonistic effects, we hoped that their insertion could optimize the activity in the NMDA receptor, leading to a dual micromolar inhibitor of both receptors.

Considering that the most promising compound, **9g**, in both previous pharmacological evaluations, P2X7R and NMDAR, featured an *o-*chloro substitution in the phenyl ring, this motif was the one selected for the right-hand side of the structure of our compounds. In addition to this, we decided to prepare **15a** due to its similarity with **3** (see Figure 2).

**Figure 3:**
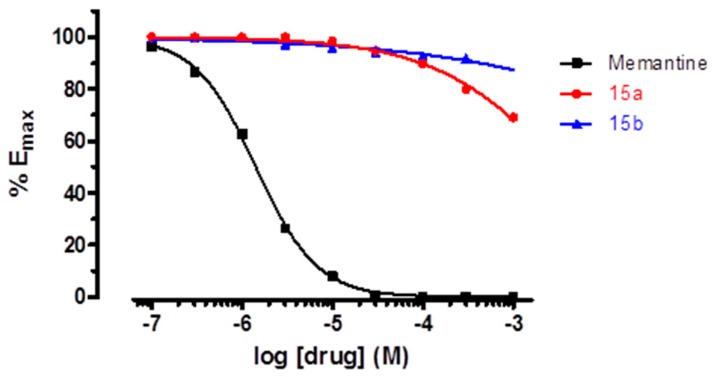
Dose–response curves for memantine and compounds **15a**–**b** in NMDA receptors.

Pleasingly, upon testing with the P2X7R, the IC_50_ of **15a** and **15b** were only slightly higher than that of **9g**, reinforcing our hypothesis that the introduction of the two-methyl groups would not be deleterious for the P2X7R antagonist activity (Table 1). However, contrary to our expectations, **15a** and **15b** were unable to significantly inhibit the NMDA receptor even at millimolar concentrations (see Figure 3 and Supplementary Material for details).

## 3. Conclusions

To summarize, we have synthesized, characterized and evaluated a series of novel adamantylcarbohydrazides as potential dual NMDAR and P2X7R antagonists. While three derivatives displayed low micromolar activities as P2X7R antagonists, only **9g** showed some activity as an NMDA receptor antagonist. Compound **9g** is, to the best of our knowledge, the first dual antagonist of both receptors.

## 4. Materials and Methods

### 4.1. Chemical Synthesis

#### 4.1.1. General Methods

Melting points were determined in open capillary tubes with an MFB 595010 M Gallenkamp. A Varian Mercury 400 spectrometer recorded 400 MHz ^1^H-NMR spectra and 100.6 MHz ^13^C-NMR spectra. The chemical shifts are reported in ppm (δ scale) relative to internal tetramethylsilane, and coupling constants are reported in hertz (Hz). Assignments given for the NMR spectra of the new compounds have been carried out on the basis of COSY ^1^H/^13^C (gHSQC and gHMBC sequences), COSY ^1^H/^1^H (standard procedures). IR spectra were run on PerkinElmer Spectrum RX I spectrophotometer (Waltham, MA, USA). Absorption values are expressed as wavenumbers (cm^−1^); only significant absorption bands are given. The GC/MS analysis was carried out in an inert Agilent Technologies 5975 gas chromatograph (Agilent Technologies Inc., Santa Clara, CA, USA) equipped with an Agilent 122-5532 DB-5MS 1b (30 m × 0.25 mm) capillary column with a stationary phase of phenylmethylsilicon (5% diphenyl–95% dimethylpolysiloxane), using the following conditions: initial temperature of 50 °C (1 min), with a gradient of 10°C/min up to 300 °C, and a temperature in the source of 250 °C, solvent delay (SD) of 4 min, and a pressure of 7.35 psi. Accurate mass measurements were obtained using the ESI technique. Absorption thin-layer chromatography was performed with aluminum-backed sheets with silica gel 60 F254 (Merck, Darmstadt, Germany, ref 1.05554), and spots were visualized with UV light and 1% aqueous solution of KMnO_4_.

#### 4.1.2. Synthesis of the Starting Acids **6** and **12** [45]

*3-Acetamidoadamantane-1-carboxylic acid.* The compound 1-Adamantane carboxylic acid, **4**, (5.0 g, 27.7 mmol) was suspended in 70% HNO_3_ (4.0 mL) and cooled at 0 °C with an ice bath. Using an addition funnel, 98% H_2_SO_4_ (30 mL) was added dropwise, at such a rate that the temperature was kept at 0 °C or below. The reaction mixture was stirred for 2 h at 0 °C, whereupon acetonitrile (20 mL) was added via an addition funnel, at such a rate that the reaction mixture was kept at 0 °C. After all the acetonitrile was added, the reaction was stirred at 0 °C for 3 h. The reaction mixture was then poured over ice (*ca.* 200 g), with shaking, and was allowed to warm to room temperature. The colorless solid was then filtered over vacuum, washed with water, and dried overnight to give the title compound (5.8 g, 88.1% yield). The ^1^H-NMR matched the data previously reported in the bibliography [46].

*3-Aminoadamantane-1-carboxylic acid hydrochloride*, **5·HCl**. The compound 3-acetamidoadamantane-1-carboxylic acid (5.8 g, 24.4 mmol), water (13.5 mL), and 37% hydrochloric acid (33.0 mL) were added to a 3-neck round bottom flask equipped with a reflux condenser, thermometer, and a mechanical stirrer; the mixture was heated at reflux for 6 days. After cooling at 0 °C with an ice bath, the white precipitate solid was carefully filtered. The solid obtained was then dried under vacuum for 1 day to afford **5·HCl** (2.6 g, 45.9% yield). The ^1^H-NMR matched the data previously reported in the bibliography [46].

*3-[(tert-Butoxycarbonyl)amino]adamantane-1-carboxylic acid*, **6**. Aqueous 2 M NaOH (10 mL) and di-*tert*-butyl dicarbonate (2.52 g, 11.7 mmol) were added to a mixture of **5·HCl** (2.6 g, 11.2 mmol) in THF (26 mL). After stirring for 16 h at room temperature, the reaction was cooled in an ice bath. Upon neutralization with aqueous 2 M HCl, the mixture was partitioned into ethyl acetate and water. The organic layer was separated, dried over anhydrous Na_2_SO_4_, filtered, and concentrated under reduced pressure to give impure **6** (2.66 g). After purification by crystallization from acetonitrile, **6** (1.5 g, 45.3% yield) was obtained as a white solid. The ^1^H-NMR matched the data previously reported in the bibliography [46].

*3-Acetamido-5,7-dimethyladamantane-1-carboxylic acid.* The compound 3,5-dimethyladamantane-1-carboxylic acid (2.0 g, 9.6 mmol) was suspended in 70% HNO_3_ (6.0 mL) and cooled to 0 °C with an ice bath. Using an addition funnel, 98% H_2_SO_4_ (10 mL) was added dropwise at such a rate that the temperature was kept at 0 °C or below. The mixture was stirred for 10 min and 20–30% oleum (3.5 mL) was added dropwise. The whole mixture was stirred for 30 min at 0 °C and 1 h at room temperature. Upon cooling again at 0 °C, acetonitrile (6.0 mL) was added and the mixture stirred for 10 min at 0 °C and 3 h at room temperature. Finally, the mixture was poured onto ice (*ca.* 200 g) and kept at 5 °C overnight. The white precipitate was collected via suction filtration and dried for 1 h to obtain the title compound as a white solid in quantitative yield [46].

*3-Amino-5,7-dimethyladamantane-1-carboxylic acid hydrochloride*, **11·HCl**. The compound 3-acetamido-5,7-dimethyladamantane-1-carboxylic acid (2.5 g, 9.42 mmol) was heated in 37% HCl (60 mL) for 5 days at reflux in a round flask equipped with a reflux condenser, thermometer, and a mechanical stirrer. After removal of the acid under reduced pressure, the crude product was washed with ethyl acetate and collected via suction filtration. The compound was dried in a desiccator over P_2_O_5_ overnight to give **11·HCl** (1.23 g, 50% yield) as a white solid. The ^1^H NMR matched the data previously reported in the bibliography [45].

*3-tert-Butoxycarbonylamino-5,7-dimethyladamantane-1-carboxylic acid*, **12**. The compound Di-*tert*-butyl dicarbonate (1.03 g, 4.73 mmol) was added to a round flask containing 11·HCl (1.23 g, 4.73 mmol) in THF (15 mL) and aqueous 2 M NaOH (6 mL). After stirring for 16 h at room temperature, the reaction was cooled into an ice bath. Following this, it was neutralized with aqueous 2 M HCl and partitioned into ethyl acetate and water. The organic layer was separated, dried over Na_2_SO_4_, filtered and concentrated under reduced pressure to give a white solid (1.15 g). The crude product was purified by crystallization from acetonitrile to give **12** (800 mg, 52% yield). The ^1^H-NMR matched the data previously reported in the bibliography [45].

#### 4.1.3. General Procedures for the Synthesis of Carbohydrazides **9a–f** or **15a–b**.

*General Procedure A.* HOBt (162 mg, 1.2 mmol) and EDC (230 mg, 1.2 mmol) were added to a suspension of **6** (295 mg, 1 mmol) or **12** (323 mg, 1 mmol) in CH_2_Cl_2_ and the whole mixture was stirred at room temperature for 2 h. The resulting mixture was then added dropwise via an addition funnel to a solution of the desired hydrazine (2 mmol, see each example for specific weights) in CH_2_Cl_2_, maintaining the temperature range between 0–10 °C. The mixture was washed with water and aqueous K_2_CO_3_, and the organic layer was dried over anhydrous Na_2_SO_4_, filtered and concentrated under reduced pressure, to yield the corresponding carbamate **8a–f** or **14a–b**.

*General Procedure B*. A solution of the corresponding crude carbamate precursor **8a–f** or **14a–b** (*ca.* 1 mmol) in CH_2_Cl_2_ (20 mL) had 4 M HCl in 1,4-dioxane (10 mL) added to it; the solution was left stirring at room temperature for 16 h. Once the reaction was completed, the mixture was extracted with aqueous 1 M HCl and the aqueous phase was then brought to basic pH with excess of aqueous 2 M NaOH. Upon extraction with CH_2_Cl_2_, the joined organic extracts were dried over anhydrous Na_2_SO_4_, filtered and concentrated under reduced pressure. The crude reaction product was crystallized from ethyl acetate, to yield the desired pure product **9a–f** or **15a–b**.

#### 4.1.4 Characterization Data for **2**, **9a–f**, and **15a–b**.

*3-Amino-N-(2-chlorobenzyl)adamantane-1-carboxamide,* **2**. Following general procedure A but using 2-chlorobenzylamine (283 mg, 2 mmol) instead of a hydrazine, the expected carbamate that was obtained was subsequently treated with the general procedure B to yield **2** as a white solid (111 mg, 35% overall yield). mp = 186–189 °C. IR (ATR): 667, 744, 868, 1036, 1049, 1261, 1287, 1359, 1408, 1439, 1467, 1529, 1628, 2139, 2842, 2899, 3333 cm^−1^. ^1^H-NMR (400 MHz, CDCl_3_) δ: 1.48 (s, 2H, NH_2_), 1.56–1.63 [complex signal, 6H, 6-H_2_ and 4(10)-H_2_], 1.67 (s, 2H, 2-H_2_), 1.72–1.80 [m, 4H, 8(9)-H_2_], 2.20 [m, 2H, 5(7)-H], 4.50 (d, *J* = 6.0 Hz, 2H, NHCH_2_), 6.05 (s, 1H, CONH), 7.19–7.25 (complex signal, 2H) and 7.31–7.37 (complex signal, 2H) (Ar-H). ^13^C-NMR (100.6 MHz, CDCl_3_) δ: 29.5 [CH, C5(7)], 35.2 (CH_2_, C6), 38.2 [CH_2_, C8(9)], 41.5 (CH_2_, NHCH_2_), 43.1 (C, C1), 45.0 [CH_2_, C4(10)], 47.81 (CH_2_, C2), 47.84 (C, C3), 127.1 (CH), 128.9 (CH), 129.5 (CH), 130.2 (CH), 133.6 (C, C2’), 135.8 (C, C1’), 176.8 (C, CO). HRMS-ESI+ *m/z* [M + H]^+^ calculated for [C_18_H_23_ClN_2_O^+^ H]^+^: 319.1572, found: 319.1574.

*3-Amino-N'-[2-(trifluoromethyl)phenyl]adamantane-1-carbohydrazide,* **9a**. Compound **8a** was obtained following general procedure A and, without further purification, **8a** was reacted with 2-(trifluoromethyl)phenylhydrazine (**7a**), 352 mg, 2 mmol) following general procedure B to give **9a** as a white solid (67 mg, 19% yield). mp = 213–216 °C. IR (ATR): 641, 695, 749, 899, 920, 1033, 1059, 1103, 1116, 1134, 1155, 1245, 1271, 1323, 1454, 1483, 1501, 1547, 1584, 1615, 1653, 2847, 2914, 3354 cm^−1^. ^1^H-NMR (400 MHz, CDCl_3_) δ: 1.45 (s, 2H, NH), 1.56–1.68 [complex signal, 6H, 6-H_2_ and 4(10)-H_2_], 1.78 (s, 2H, 2-H_2_), 1.82–1.92 [m, 4H, 8(9)-H_2_], 2.26 [m, 2H, 5(7)-H], 6.56 (s, 1H, NH), 6.93 (d, *J* = 7.6 Hz, 1H, 6’-H), 6.96 (d, *J* = 7.6 Hz, 1H, 4’-H), 7.39 (t, *J* = 7.6 Hz, 1H, 5’-H), 7.51 (d, *J* = 8.4 Hz, 1H, 3’-H). ^13^C-NMR (100.6 MHz, CDCl_3_) δ: 29.4 [CH, C5(7)], 35.0 (CH_2_, C6), 37.9 [CH_2_, C8(9)], 42.6 (C, C1), 44.9 [CH_2_, C4(10)], 47.4 (CH_2_, C2), 47.7 (C, C3), 113.2 (CH, C6’), 115.3 (q, *J* = 29.5 Hz , C, C2’), 120.3 (C, C4’), 124.5 (q, *J* = 269.3 Hz, C, CF_3_), 126.6 (q, *J* = 5.4 Hz, CH, C3’), 132.9 (CH, C5’), 145.9 (C, C1’), 176.6 (C, CO). HRMS-ESI+ *m*/*z* [M + H]^+^ calculated for [C_18_H_22_F_3_N_3_O^+^ H]^+^: 354.1788, found: 354.1794.

*3-Amino-N'-(2,6-dichlorophenyl)adamantane-1-carbohydrazide,* **9b**. Compound **8b** was obtained following general procedure A and, without further purification, **8b** was reacted with 2,6-dichlorophenylhydrazine (**7b**, 354 mg, 2 mmol) following general procedure B to give **9b** as a colourless solid (135 mg, 38% yield). mp = 153–156 °C. IR (ATR): 713, 760, 863, 935, 1085, 1304, 1418, 1444, 1524, 1576, 1594, 1653, 2020, 2847, 2914, 2930, 3136, 3322 cm^−1^. ^1^H-NMR (400 MHz, CDCl_3_) δ: 1.25 (broad signal, 2H, NH_2_), 1.50–1.62 [complex signal, 6H, 6-H_2_ and 4(10)-H_2_], 1.68 (s, 2H, 2-H_2_), 1.71–1.82 [complex signal, 4H, 8(9)-H_2_], 2.19 [m, 2H, 5(7)-H], 6.78 (d, *J* = 4.8 Hz, 1H, NH), 6.88 (t, *J* = 8.9 Hz, 1H, 4’-H), 7.23 [d, *J* = 8.0 Hz, 2H, 3’(5’)-H], 7.92 (d, *J* = 5.2 Hz, 1H, NH). ^13^C-NMR (100.6 MHz, CDCl_3_) δ: 29.4 [CH, C5(7)], 35.1 (CH_2_, C6), 37.8 [CH_2_, C8(9)], 42.7 (C, C1), 45.0 [CH_2_, C4(10)], 47.5 (CH_2_, C2), 47.6 (C, C3), 123.9 (CH, C4’), 125.8 [C, C2’(6’)], 128.9 [CH, C3’(5’)], 141.3 (C, C1’), 175.4 (C, CO). HRMS-ESI+ *m/z* [M + H]^+^ calculated for [C_17_H_21_Cl_2_N_3_O^+^ H]^+^: 354.1134, found: 354.1140.

*3-Amino-N'-(2,3-dichlorophenyl)adamantane-1-carbohydrazide,* **9c**. Compound **8c** was obtained following general procedure A and, without further purification, **8c** was reacted with 2,3-dichlorophenylhydrazine (**7c**, 354 mg, 2 mmol) following general procedure B to give **9c** as a light yellow solid (127 mg, 36% yield). mp = 215–219 ^o^C. IR (ATR): 767, 912, 938, 1041, 1106, 1137, 1170, 1266, 1287, 1421, 1449, 1491, 1578, 1648, 2842, 2894, 3348 cm^−1^. ^1^H-NMR (400 MHz, DMSO) δ: 1.42–1.57 [complex signal, 6H, 6-H_2_ and 4(10)-H_2_], 1.63 (s, 2H, 2-H_2_), 1.70–1.80 [complex signal, 4H, 8(9)-H_2_], 2.10 [s, 2H, 5(7)-H], 6.61 (dd, *J* = 8.0 Hz, *J’* = 1.6 Hz, 1H, 6’-H), 6.95 (dd, *J* = 8.0 Hz, *J’* = 1.6 Hz, 1H, 4’-H), 7.15 (t, *J* = 8.0 Hz, 1H, 5’-H), 7.58 (s, 1H, NH). ^13^C-NMR (100.6 MHz, DMSO) δ: 29.3 [CH, C5(7)], 35.2 (CH_2_, C6), 37.7 [CH_2_, C8(9)], 41.9 (C, C1), 44.9 [CH_2_, C4(10)], 47.3 (CH_2_, C2), 47.6 (C, C3), 111.0 (CH, C6’), 115.1 (C, C3’), 119.5 (CH, C4’), 128.4 (CH, C5’), 131.8 (C, C2’), 147.2 (C, C1’), 176.1 (C, CO). HRMS-ESI+ *m/z* [M + H]^+^ calculated for [C_17_H_21_Cl_2_N_3_O^+^ H]^+^: 354.1134, found: 354.1140.

*3-Amino-N'-[2-chloro-4-(trifluoromethyl)phenyl]adamantane-1-carbohydrazide,* **9d**. Compound **8d** was obtained following general procedure A and, without further purification, **8d** was reacted with 2-chloro-4-(trifluromethyl)phenylhydrazine (**7d**, 421 mg, 2 mmol) following general procedure B to give **9d** as a light orange solid (54 mg, 14% yield). mp = 185–186 °C. IR (ATR): 659, 822, 881, 899, 930, 1046, 1111, 1256, 1416, 1491, 1578, 1612, 1653, 2899, 2925, 3198, 3385, 3627 cm^−1^. ^1^H-NMR (400 MHz, DMSO) δ: 1.40–1.60 [complex signal, 6H, 6-H_2_ and 4(10)-H_2_], 1.64 (s, 2H, 2-H_2_), 1.70–1.88 [complex signal, 4H, 8(9)-H_2_], 2.11 [s, 2H, 5(7)-H], 6.81 (d, *J* = 8.0 Hz, 1H, 6’-H), 7.45–7.60 (complex signal, 2H, 3’-H and 5’-H), 7.56 (broad s, 1H, NH). ^13^C-NMR (100.6 MHz, DMSO) δ: 29.2 [CH, C5(7)], 35.2 (CH_2_, C6), 37.7 [CH_2_, C8(9)], 41.9 (C, C1), 44.9 [CH_2_, C4(10)], 47.2 (CH_2_, C2), 47.6 (C, C3), 112.8 (q, *J* = 31.0 Hz, C, C4’), 114.9 (CH, C6’), 121.4 (C, C2’), 123.8 (q, *J* = 273.0 Hz, CF_3_), 125.8 (broad signal, CH, C3’), 133.3 (CH, C5’), 145.8 (C, C1’), 176.1 (C, CO). HRMS-ESI+ *m/z* [M + H]^+^ calculated for [C_18_H_21_Cl F_3_N_3_O^+^ H]^+^: 388.1398, found: 388.1404.

*3-Amino-N'-(3,5-dichloropyridin-4-yl)adamantane-1-carbohydrazide,* **9e**. Compound **8e** was obtained following general procedure A and, without further purification, **8e** was reacted with 3,5-dichloro-4-hydrazinopyridine (**7e**, 356 mg, 2 mmol) following general procedure B to give **9e** as a white solid (106 mg, 30% yield). mp = 191–192 ^o^C. IR (ATR): 729, 793, 886, 935, 1000, 1083, 1090, 1225, 1279, 1395, 1444, 1475, 1540, 1558, 1589, 1659, 1958, 1997, 2077, 2153, 2206, 2341, 2909, 3173 cm^−1^. ^1^H-NMR (400 MHz, CDCl_3_) δ: 1.25–1.42 (broad s, 2H, NH), 1.54–1.63 [complex signal, 6H, 6-H_2_ and 4(10)-H_2_], 1.71 (s, 2H, 2-H_2_), 1.74–1.85 [complex signal, 4H, 8(9)-H_2_], 2.23 [m, 2H, 5(7)-H], 6.81 (s, 1H, NH), 7.87 (s, 1H, NH), 8.29 [s, 2H, 2’(6’)-H]. ^13^C-NMR (100.6 MHz, CDCl_3_) δ: 29.4 [CH, C5(7)], 35.0 (CH_2_, C6), 37.8 [CH_2_, C8(9)], 42.8 (C, C1), 44.9 [CH_2_, C4(10)], 47.4 (CH_2_, C2), 47.6 (C, C3), 120.6 [C, C3’(5’)], 147.3 (C, C4’), 148.4 [CH, C2’(6’)], 175.5 (C, CO) HRMS-ESI+ *m/z* [M + H]^+^ calculated for [C_16_H_20_Cl_2_N_4_O^+^ H]^+^: 355.1087, found: 355.1094.

*3-Amino-N'-(3-chloro-2-fluorophenyl)adamantane-1-carbohydrazide,* **9f**. Compound **8f** was obtained following general procedure A and, without further purification, **8f** was reacted with 3-chloro-2-flurophenylhydrazine (**7f**, 321 mg, 2 mmol) following general procedure B to give **9f** as a yellowish solid (84 mg, 25% yield). mp = 200–202 °C. IR (ATR): 708, 762, 814, 920, 935, 943, 1044, 1093, 1124, 1219, 1284, 1457, 1470, 1506, 1584, 1607, 1648, 1984, 2847, 2899, 3307 cm^−1^. ^1^H-NMR (400 MHz, DMSO) δ: 1.00–1.42 [complex signal, 6H, 6-H_2_ and 4(10)-H_2_], 1.62 (s, 2H, 2-H_2_), 1.68–1.78 [complex signal, 4H, 8(9)-H_2_], 2.10 [broad s, 2H, 5(7)-H], 6.61 (t, *J* = 8.0 Hz, 1H, 6’-H), 6.82 (t, *J* = 7.6 Hz, 1H, 4’-H), 6.99 (t, *J* = 7.6 Hz, 1H, 5’-H), 7.83 (s, 1H, NH). ^13^C-NMR (100.6 MHz, DMSO) δ: 29.3 [CH, C5(7)], 35.2 (CH_2_, C6), 37.7 [CH_2_, C8(9)], 41.9 (C, C1), 44.8 [CH_2_, C4(10)], 47.2 (CH_2_, C2), 47.7 (C, C3), 112.1 (CH, C6’), 118.7 (CH, C4’), 119.5 (d, *J* = 14.6 Hz, CH, C3’), 125.2 (d, *J* = 4.2 Hz, CH. C5’), 139.9 (d, *J* = 10.2 Hz, C, C1’), 145.6 (d, *J* = 241.5 Hz, C, C2’), 176.2 (C, CO). HRMS-ESI+ *m/z* [M + H]^+^ calculated for [C_17_H_21_ClFN_3_O^+^ H]^+^: 338.1430, found: 338.1439.

*3-Amino-N'-(2-chlorophenyl)adamantane-1-carbohydrazide*, **9g**. Compound **8g** was obtained following general procedure A and, without further purification, **8g** was reacted with 2-chlorophenylhydrazine (**7g**, 285 mg, 2 mmol) following general procedure B to give **9g** as a pale orange solid (90 mg, 28% yield). mp = 220–222 ^o^C. IR (ATR): 734, 902, 925, 1033, 1046, 1292, 1442, 1496, 1545, 1586, 1594, 1646, 2904, 2925, 3322 cm^−1^. ^1^H-NMR (400 MHz, CDCl_3_) δ: 1.42 (broad s, 2H, NH), 1.56-1.67 [complex signal, 6H, 6-H_2_ and 4(10)-H_2_], 1.75 (s, 2H, 2-H_2_), 1.76–1.87 [complex signal, 4H, 8(9)-H_2_], 2.23 [s, 2H, 5(7)-H], 6.43 (broad s, 1H, NH), 6.80 (dd, *J* = 8.0 Hz, *J’* = 1.6 Hz, 1H, 6’-H), 6.83 (td, *J* = 8.0 Hz, *J’* = 1.6 Hz, 1H, 4’-H), 7.13 (td, *J* = 8.0 Hz, *J’* = 1.6 Hz, 1H, 5’H), 7.27 (dd, *J* = 8.0 Hz, *J’* = 1.6 Hz, 3’H), 7.54 (broad s, 1H, NH). ^13^C-NMR (100.6 MHz, CDCl_3_) δ: 29.4 [CH, C5(7)], 35.1 (CH_2_, C6), 37.9 [CH_2_, C8(9)], 42.6 (C, C1), 44.9 [CH_2_, C4(10)], 47.5 (CH_2_, C2), 47.7 (C, C3), 113.4 (CH, C6’), 119.9 (C, C2’), 121.5 (CH, C4’), 127.6 (CH, C5’), 129.5 (CH, C3’), 144.1 (C, C1’), 176.5 (C, CO). HRMS-ESI+ *m/z* [M + H]^+^ calculated for [C_17_H_22_ClN_3_O^+^ H]^+^: 320.1524, found: 320.1528.

*3-Amino-N'-(3,5-dichloropyridin-4-yl)-5,7-dimethyladamantane-1-carbohydrazide,* **15a**. Crude **14a** was obtained from **12** (323 mg, 1 mmol) following general procedure A. Without further purification, **14a** was reacted with 3,5-dichloro-4-hydrazinopyridine **13a** (356 mg, 2 mmol) following general procedure B to give **15a** as a white solid (96 mg, 25% yield). mp = 300–302 °C. ^1^H NMR (400 MHz, CDCl_3_) δ: 0.91 (s, 6H, CH_3_), 1.12 (broad s, 2H, 6-H_2_), 1.20-1.27 [complex signal, 4H, 4(10)-H_2_], 1.42–1.48 [complex signal, 4H, 8(9)-H_2_], 1.58 (s, 2H, 2-H_2_), 6.80 (s, 1H, NH), 7.93 (s, 1H, NH), 8.29 [s, 2H, 2’(6’)-H]. ^13^C-NMR (100.6 MHz, CDCl_3_) δ: 29.6 (CH_3_, CH_3_), 32.9 [C, C5(7)], 44.1 [CH_2_, C8(9)], 44.2 (C, C1), 46.1 (CH_2_, C2), 49.3 (C, C3), 49.5 (CH_2_, C6), 51.4 [CH_2_, C4(10)], 120.6 [CH, C3’(5’)], 147.2 (C, C4’), 148.3 [CH, C2’(6’)], 175.4 (C, CO). HRMS-ESI+ *m/z* [M + H]^+^ calculated for [C_18_H_24_Cl_2_N_4_O^+^ H]^+^: 383.1400, found: 383.1404.

*3-Amino-N'-(2-chlorophenyl)-5,7-dimethyladamantane-1-carbohydrazide*, **15b**. Crude **14b** was obtained from **12** (323 mg, 1 mmol) following general procedure A. Without further purification, **14b** was reacted with 2-chlorophenylhydrazine **13b** (285 mg, 2 mmol) following general procedure B to give **15b** as a white solid (97 mg, 28% yield). mp = 280–282 °C. IR (ATR): 646, 736, 1031, 1230, 1289, 1356, 1390, 1449, 1540, 1648, 2480, 2857, 2930, 3245, 3297 cm^-1^. ^1^H NMR (400 MHz, CD_3_OD) δ: 0.96 (s, 6H, CH_3_), 1.19 (broad s, 2H, 6-H_2_), 1.29–1.37 [complex signal, 4H, 4(10)-H_2_], 1.55 [broad s, 4H, 8(9)-H_2_], 1.69 (s, 2H, 2-H_2_), 6.76–6.82 (complex signal, 2H, 4’-H and 6’-H), 7.15 (dt, *J* = 8.0 Hz, *J’* = 1.6 Hz, 1H, 5’-H), 7.25 (dd, *J* = 8.0 Hz, *J’* = 1.6 Hz, 1H, 3’-H). ^13^C-NMR (100.6 MHz, CD_3_OD) δ: 30.1 (CH_3_, CH_3_), 33.9 [C, C5(7)], 45.0 (C, C1), 45.1 [CH_2_, C8(9)], 45.4 (CH_2_, C2), 50.5 (CH_2_, C6), 50.6 [CH_2_, C4(10)], 51.1 (CH_2_, C3), 114.2 (CH, C6’), 120.1 (C, C2’), 121.5 (CH, C4’), 128.7 (CH, C5’), 130.3 (CH, C3’), 145.9 (C, C1’), 178.8 (C, CO). HRMS-ESI+ *m/z* [M + H]^+^ calculated for [C_19_H_26_ClN_3_O^+^ H]^+^: 348.1764, found: 348.1801.

### 4.2. P2X7 Receptor Antagonist Activity. Dye Uptake Using the Ethidium Ion Assay

HEK293 cells stably expressing the P2X7 receptor were maintained in Dulbecco's modified Eagle's medium (DMEM) supplemented with 10% fetal bovine serum (FBS), 2 mM l-glutamine, and antibiotics (50 U/mL penicillin and 50 mg/mL streptomycin) in a humidified 5% CO_2_ atmosphere at 37 °C. We used Lipofectamine as a transfection reagent with a pcDNA3.1 vector-based plasmid harbouring *h*P2X7R (Invitrogen, Waltham, MA, USA). After diluting to 2.5 × 10^6^ cells/mL, a buffer composed of 10 mM HEPES, 5 mM *N*-methyl-d-glutamine, 5.6 mM KCl, 10 mM d-glucose, and 0.5 mM CaCl_2_ (pH 7.4), supplemented with either 280 mM sucrose or 140 mM NaCl and an 80 μL aliquot, was added to each well of 96-well culture plates. The test compounds and 2(3’)-*O*-(4-benzoylbenzoyl)-ATP (BzATP) were then added, and the cells were incubated for 2 h in a humidified 5% CO_2_ atmosphere at 37 °C. After incubation, a Bio-Tek FL600 fluorescence plate reader (Winooski, VT, USA) was used to measure the absorbance at an excitation wavelength of 530 nm and an emission wavelength of 590 nm. The inhibition (percent) of ethidium ion uptake was expressed as a relative value of the maximum accumulation when stimulated with BzATP only. To calculate IC_50_ values, we calculated a series of dose–response data using nonlinear regression analysis (i.e., percentage accumulation of ethidium bromide *vs* compound concentration). The concentration of ethidium bromide was 0.1 mM.

### 4.3. NMDA Receptor Antagonist Activity

The functional assay of antagonist activity at the NMDA receptors was performed using primary cultures of rat cerebellar granule neurons that were prepared according to established protocols [48]. Cells were grown on 10 mm poly-l-lysine coated glass cover slips, and used for the experiments after 6–9 days in vitro. Cells were loaded with 6 µM Fura-2 AM (ThermoFisher Scientific-Molecular Probes) for 30 min. Afterwards, the coverslip was mounted on a quartz cuvette containing a Locke-Hepes buffer using a special holder. Measurements were performed using a PerkinElmer LS-55 fluorescence spectrometer (Waltham, MA, USA) equipped with a fast-filter accessory, under mild agitation and at 37 °C. Analysis of each sample was recorded in real time for 1600 s. After stimulation with NMDA (100 µM, in the presence of 10 µM glycine), increasing cumulative concentrations of the compound to be tested were added. The percentages of inhibition at every tested concentration were analyzed using a non-linear regression curve fitting analysis by using the software GraphPad Prism 5.0 (GraphPad Software Inc., La Jolla, CA, USA).

## Figures and Tables

**Table 1:** Antagonistic activity in *h*P2X7-expressing HEK293 cells ^a^.

Compound	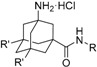		% Inhibition or IC_50_^a,b^
	R	R’	
no BzATP	-	-	0%
only BzATP	-	-	100%
AZD9056 ^c^	-	-	4.1 ± 0.7 nM ^d^
**2**	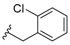	H	NA ^e^
**9a**	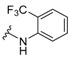	H	48%/37%
**9b**	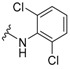	H	23%/16%
**9c**	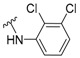	H	42%/17%
**9d**	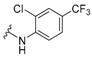	H	24%/21%
**9e**	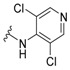	H	53%/27%
**9f**	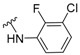	H	15%/17%
**9g**	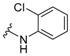	H	6.0 ± 0.6 μM ^d^
**15a**	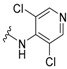	CH_3_	9.9 ± 0.7 μM ^d^
**15b**	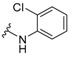	CH_3_	16.1 ± 1.6 μM ^d^

^a^ Percentage inhibition values at 10 µM (left) and 1 μM (right) were expressed as percentages, relative to the maximum uptake of ethidium bromide stimulated by BzATP 6 µM. All experiments were repeated at least 3–6 times. ^b^ Experiments were assessed in the ethidium bromide (EtBr) accumulation assay using *h*P2X7-expressing human embryonic kidney 293 (HEK293) cells. ^c^ Positive control [46]. ^d^ IC_50_ value was obtained from concentration–response curves. ^e^ Not active.

## Supplementary Materials

Supplementary materials can be accessed online.
