# Supplementary material for: Towards a Novel Class of Multitarget-Directed Ligands: Dual P2X7–NMDA Receptor Antagonists
Source: Molecules. 2018 Jan 21;23(1):230. doi: 10.3390/molecules23010230 (PMC6017257; doi:10.3390/molecules23010230)
Supplement: Supplementary file 1 [file molecules-23-00230-s001.pdf]

**SUPPLEMENTARY MATERIAL for**

# Towards a Novel Class of Multitarget Directed Ligands: Dual P2X7 – NMDA Receptors Antagonists

Olga Karoutzou, Seung-Hwa Kwak, So-Deok Lee, Daina Martínez-Falguera, Francesc X. Sureda, Santiago Vázquez, Yong-Chul Kim* and Marta Barniol-Xicota*

## INDEX

**Figures S1-S4**. Dose-response curves for antagonist compounds AZD 9056, **9g** and **15a-b** in hP2X7-expressing HEK293 cells. S2

**Table S1**. NMDA antagonist activity in rat cerebellar granule cells. S4

**Figures S1-S4**. Dose-response curves for antagonist compounds AZD 9056, **9g** and **15a-b** in hP2X7-expressing HEK293 cells.

**AZD 9056** $IC_{50}$ = 4.1 nM

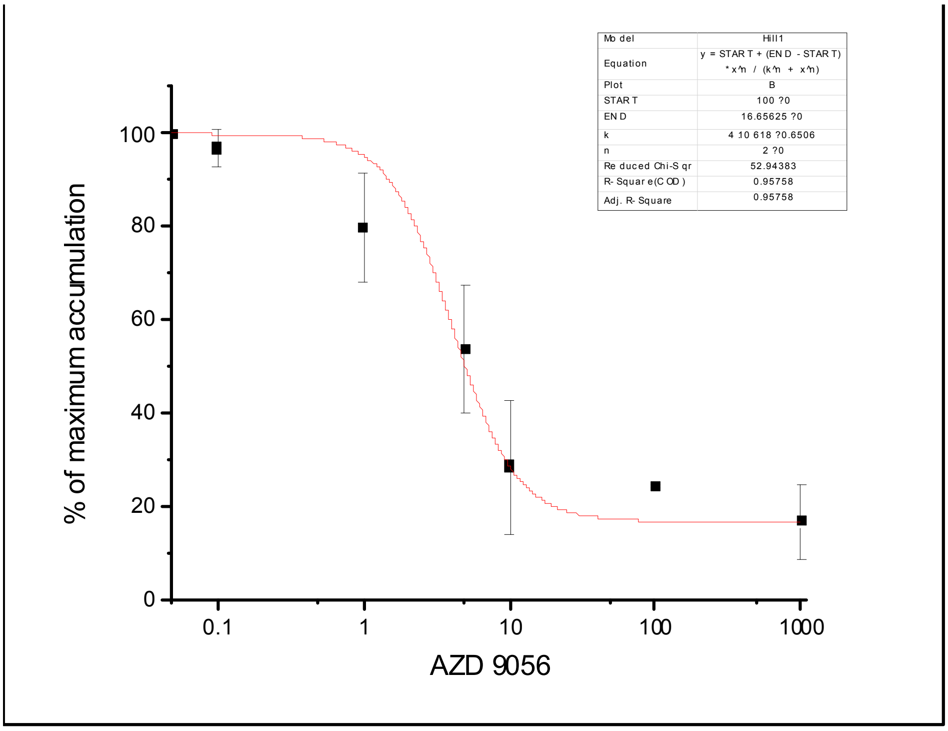


Compound **9g** $IC_{50}$ = 6 µM

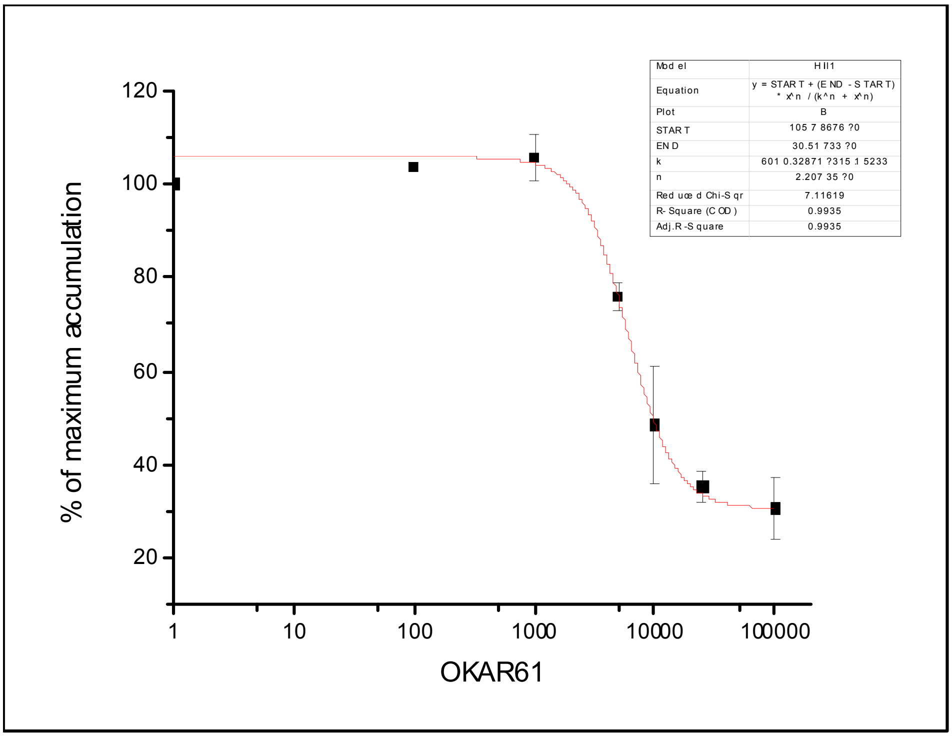

Compound **15a** $IC_{50}$ = 9.9 μM

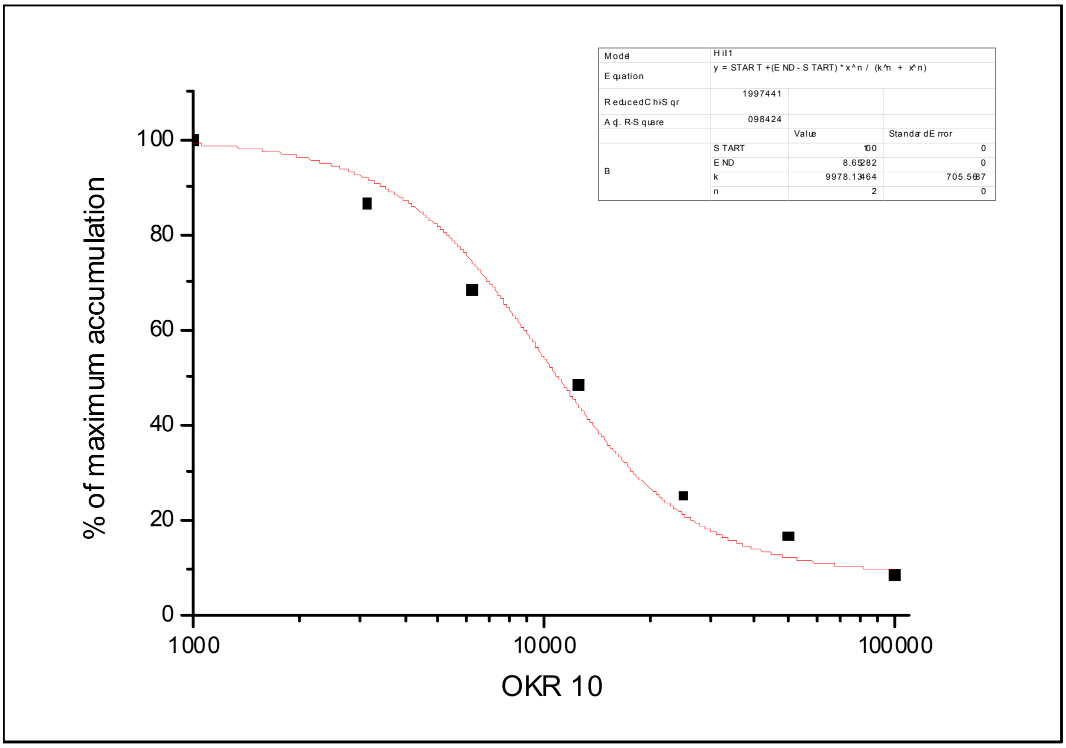


Compound **15b** $IC_{50}$ = 16.1 μM

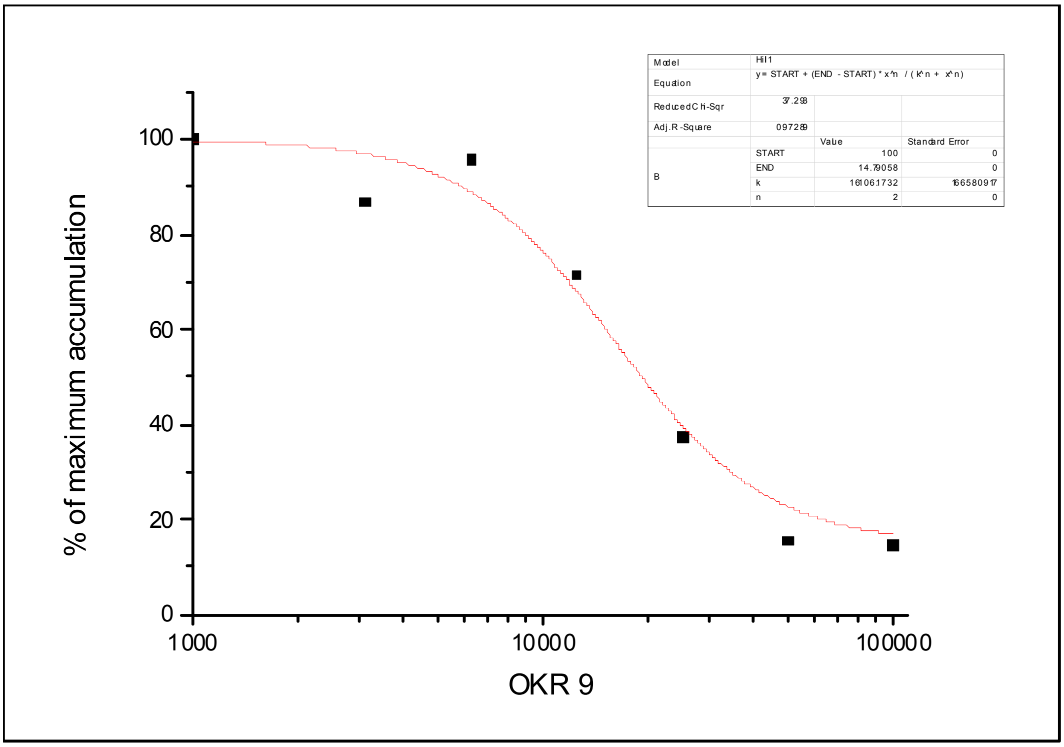

**Table S1.** NMDA antagonist activity in rat cerebellar granule cells.

| Compound | 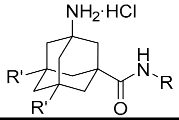 R | R′ | % inhibition[a] or $IC_{50}$[b] |
|---|---|---|---|
| **2** | 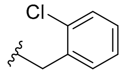 | H | 18% / 30% |
| **9a** | 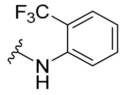 | H | 5% / 14% |
| **9b** | 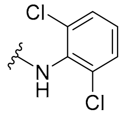 | H | 17% / 38% |
| **9c** | 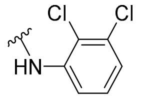 | H | 23% / 30% |
| **9d** | 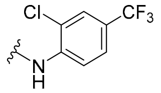 | H | 20% / Not assayed |
| **9e** | 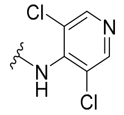 | H | 6% / 16% |
| **9f** | 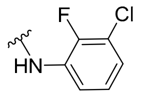 | H | 11% / 18% |
| **9g** | 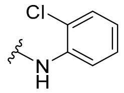 | H | 468 ± 23 |
| **15a** | 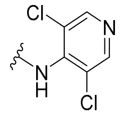 | $CH_3$ | 20% / 31% |
| **15b** | 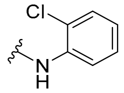 | $CH_3$ | 7% / 8% |

[a]Percentage inhibition values at 100 µM (left) and 300 µM (right) were expressed as percentages, relative to maximum intracellular calcium concentration stimulated by 100 µM NMDA in the presence of 10 µM of glycine. All experiments were repeated at least three times. [b]$IC_{50}$ value was obtained from concentration response curves.
